# Optoelectronic Synapses Based on MXene/Violet Phosphorus van der Waals Heterojunctions for Visual-Olfactory Crossmodal Perception

**DOI:** 10.1007/s40820-024-01330-7

**Published:** 2024-02-01

**Authors:** Hailong Ma, Huajing Fang, Xinxing Xie, Yanming Liu, He Tian, Yang Chai

**Affiliations:** 1 Center for Advancing Materials Performance From the Nanoscale (CAMP-Nano), State Key Laboratory for Mechanical Behavior of Materials, Xi’an Jiaotong University, Xi’an, 710049 People’s Republic of China; 2 Institute of Microelectronics and Beijing National Research Center for Information Science and Technology (BNRist), Tsinghua University, Beijing, 100084 People’s Republic of China; 3 Department of Applied Physics, The Hong Kong Polytechnic University, Hong Kong, People’s Republic of China

**Keywords:** Violet phosphorus, MXene, Van der Waals heterojunctions, Optoelectronic synapses, Crossmodal perception

## Abstract

The crossmodal interaction of different senses, which is an important basis for learning and memory in the human brain, is highly desired to be mimicked at the device level for developing neuromorphic crossmodal perception, but related researches are scarce. Here, we demonstrate an optoelectronic synapse for vision-olfactory crossmodal perception based on MXene/violet phosphorus (VP) van der Waals heterojunctions. Benefiting from the efficient separation and transport of photogenerated carriers facilitated by conductive MXene, the photoelectric responsivity of VP is dramatically enhanced by 7 orders of magnitude, reaching up to 7.7 A W^−1^. Excited by ultraviolet light, multiple synaptic functions, including excitatory postsynaptic currents, paired-pulse facilitation, short/long-term plasticity and “learning-experience” behavior, were demonstrated with a low power consumption. Furthermore, the proposed optoelectronic synapse exhibits distinct synaptic behaviors in different gas environments, enabling it to simulate the interaction of visual and olfactory information for crossmodal perception. This work demonstrates the great potential of VP in optoelectronics and provides a promising platform for applications such as virtual reality and neurorobotics.

**Supplementary Information:**

The online version contains supplementary material available at 10.1007/s40820-024-01330-7.

## Highlights

- The photoelectric response of violet phosphorus (VP) is significantly enhanced by the MXene/VP van der Waals heterojunctions and the highest photoresponsivity of VP is demonstrated.
- The first VP-based optoelectronic synapse with essential synaptic behaviours is demonstrated.
- Proof-of-concept visual-olfactory crossmodal perception based on MXene/VP optoelectronic synapses is explored to mimic neuromorphic vision with multi-sensory interactions.

**Supplementary Information:**

The online version contains supplementary material available at 10.1007/s40820-024-01330-7.

## Graphical abstract

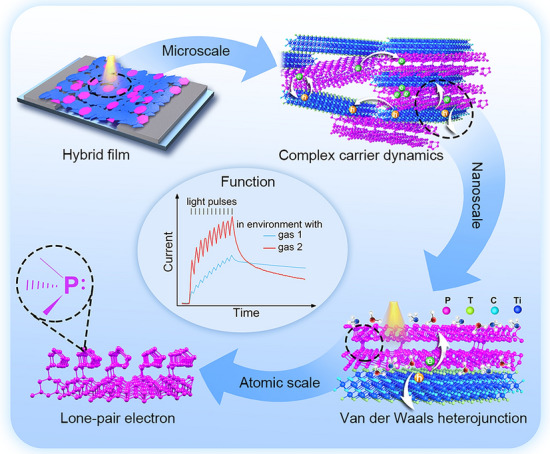

## Introduction

With diverse senses such as vision, olfaction, touch, hearing, vestibular sense as well as their crossmodal interactions, human beings acquire environmental information and respond accordingly after being processed by the brain [1–4]. Inspired by human multi-sensory systems, neuromorphic sensing and computing systems with biomimetic sensors and neural networks have been explored to sense and process various sensory information and their crossmodal integration [5–8]. Given that over eighty percent of the information that humans perceive from the environment is obtained through the visual system, optoelectronic synapses have been proposed to simulate brain-like visual perception in recent years [9–12]. Unlike conventional imaging sensors that only respond linearly to light intensity, optoelectronic synapses not only directly respond to light stimuli, but also allow temporary processing and memory of visual information at each pixel, promising more efficient and energy-saving artificial vision systems [13]. Furthermore, crossmodal perception such as visual-tactile and visual-auditory interactions based on optoelectronic synapses and neural networks has been explored for more realistically simulating neuromorphic vision [2, 4, 6, 14, 15]. Neuroscience and psychology studies have proven that the human brain also has a crossmodal interaction between visual and olfactory perception [1, 3, 16, 17]. For example, Zhou et al. found that olfaction warps visual time perception by recording electroencephalogram stimulated by rapidly flashing fruit pictures and corresponding odors [18]. However, relevant researches at the device level are absent. Therefore, it is highly desirable to explore optoelectronic synapses with visual-olfactory crossmodal perception for mimicking neuromorphic vision with multi-sensory interactions.

To this end, rational employment of materials is required since the neuromorphic functionalities strongly depends on material properties [19]. Extensive investigations have shown that Van der Waals heterojunctions based on two-dimensional (2D) materials can serve as excellent building blocks for optoelectronic synapses due to the engineered manipulation of photogenerated excitons and carrier transport at the interface [20–24]. Moreover, the combination of the unique properties of each 2D component enables them to sense various environmental signals such as light, pressure, temperature and chemical molecules, which holds promise for optoelectronic synapses with crossmodal perception [25–27]. Among the diverse 2D materials, violet phosphorus (VP), as an emerging 2D layered semiconductor, has a calculated high carrier mobility (3000–7000 cm^2^ V^−1^ s^−1^), moderate band gap (2.5 eV) and high Young’s modulus [28, 29]. Additionally, the huge specific surface area and the unique electronic structure with lone pairs electrons provide natural active sites, endowing VP with high sensitivity to changing gas environments [30]. These attractive properties make VP a promising candidate for crossmodal optoelectronic synapses. However, there are only a few reports on its photoelectric response, with the maximum responsivity of 10 mA W^−1^ [31–33]. The below-expected responsivity is attributed to the poor separation of photogenerated electron–hole (e–h) pairs caused by the high exciton binding energy and strong radiative recombination of VP [29, 33, 34]. Constructing van der Waals heterostructures with other 2D materials to promote the separation of photogenerated e–h pairs is an optional way to improve the optoelectronic performance of VP [35], but related research is quite scarce.

As a new branch of 2D materials, transition-metal carbides and nitrides (MXenes) are promising candidates to address above challenges in view of their extraordinary properties such as high conductivity, tunable work function, and good hydrophilicity [36–38]. In this work, we demonstrate the highest photoelectric responsivity of VP and the first VP-based crossmodal optoelectronic synapse. Specifically, we fabricated a hybrid film composed of van der Waals heterojunctions based on MXene (Ti_3_C_2_T_*x*_, T stands for the surface terminations such as –O, –OH, and –F) and VP nanosheets through a simple but robust solution process. The responsivity of MXene/VP heterojunctions is as high as 7.7 A W^−1^ under 360 nm ultraviolet (UV) light, which is 7 orders of magnitude higher than that of pure VP. The significantly enhanced photoelectric response is attributed to the efficient separation and transport of photogenerated carriers of VP facilitated by MXene. Based on the persistent photoconductivity (PPC) effect of MXene/VP heterojunction network, we demonstrate an optoelectronic synapse with multiple synaptic behaviors. Furthermore, the device exhibits distinct photoelectric responses as well as image learning and memory properties in different gas environments, enabling it to work as a photoelectric synapse with visual-olfactory crossmodal perception. This work demonstrates the promising application of VP in optoelectronics and provides a paradigm for crossmodal optoelectronic synapses based on 2D materials.

## Experimental Section

### Materials Synthesis

Ti_2_C_3_T_x_ MXene nanosheets were prepared by chemically etching the Al layers from purchased Ti_3_AlC_2_ powder (Jilin 11 Technology Co., Ltd.), followed by ultrasonic treatment. Specifically, 1.6 g LiF was added to 20 mL HCl (12 M) and stirred until dissolved. Then, 1 g Ti_3_AlC_2_ powder was slowly added to the above mixed solution and stirred in an oil bath at 40 °C for 24 h to completely etch the Al layers. Next, the reactants were washed by centrifugation with deionized water until almost neutral (pH ≥ 6). Subsequently, deionized water was added to the resulting sediment and sonicated in an ice bath for one hour to exfoliate the multilayer MXene. Finally, the mixture was centrifuged at 3500 rpm for 1 h and the supernatant containing single or few layers of MXene nanosheets was collected. The concentration of MXene colloidal solution was determined to be 6 mg mL^−1^ by weighing the MXene film obtained by suction filtration. Violet Phosphorus dispersion (0.2 mg mL^−1^) was purchased from Nanjing XFNANO Materials Tech Co., Ltd.

### Device Fabrication

The prepared MXene colloidal solution was diluted to 0.6 mg mL^−1^ with deionized water, and mixed with violet phospholene dispersion at a volume ratio (mL) of 0.01:1, 0.02:1 and 0.03:1, respectively. The resulting mixture was placed on a vortex mixer to mix it thoroughly. Au electrodes with a thickness of 60 nm were thermally evaporated onto poly(vinylidene fluoride) (PVDF) filter paper using a patterned shadow mask. The length and width of the channel are 50 and 2000 μm respectively. Then, the mixture of MXene and violet phospholene was coated onto the channel by suction filtration. Finally, the obtained device was dried in a nitrogen glove box at 100 °C for 10 min.

### Characterization

The morphology of the sample was characterized by scanning electron microscope (SEM, Zeiss Gemini500) and transmission electron microscope (TEM, JEOL JEM-F200). Raman spectrum was obtained by a Raman spectroscopy (LabRAM HR Evolution) equipped with a 532 nm laser. The chemical compositions and work functions of the samples were analyzed through X-ray photoelectron spectroscopy (XPS, Thermo Fisher ESCALAB Xi +). The steady-state PL spectroscopy tests were performed by a transient steady-state fluorescence spectrometer (Edinburgh FLS9) with a 532 nm laser. Environments with different gas atmospheres and RH were created by placing corresponding volatile liquids and saturated salt solutions in sealed test chambers. All electrical tests were performed through a digital source meter (Keithley 2410). Two lasers with wavelengths of 360 nm and 532 nm were used as illumination sources and the light power was calibrated by a NOVA II power meter (OPHIR photonics, Israel). The light pulse signal was generated by an electronic timer (GCI-73).

## Results and Discussion

### Multiscale Structural Design and Characterization

**Fig. 1:**
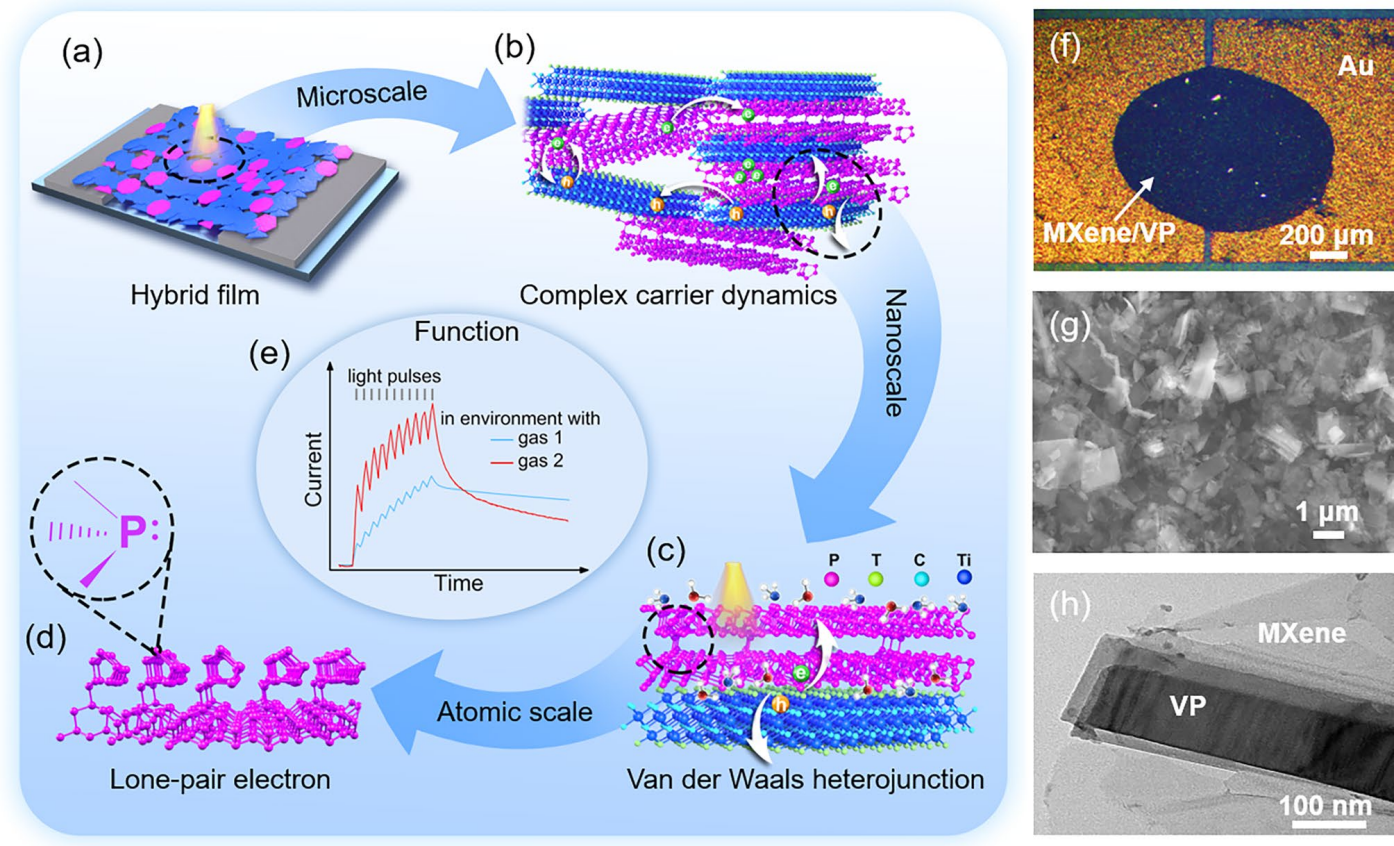
**a** Schematic illustration of the device configuration. **b** Schematic diagram of complex carrier dynamics processes such as separation, hopping transport, trapping and recombination of photogenerated carriers in MXene/VP heterojunctions network. **c** Schematic diagram of the MXene/VP van der Waals heterojunction. **d** Atomic structure of VP and lone-pair electrons of P atom. **e** Output characteristics of the visual-olfactory crossmodal optoelectronic synapse. **f** Optical microscope image of the device. **g** SEM image of the MXene/VP hybrid film. **h** TEM image of a single MXene/VP heterojunction

The device function of visual-olfactory crossmodal perception is manifested by different synaptic behaviors in different gas environments excited by light pulses, which requires rational material selection and device design at the begining. Figure 1 presents an overall schematic diagram of the multiscale design route that focuses on device function. Figure 1a is the designed device configuration, in which a hybrid film composed of abundant MXene/VP heterostructures are suction filtered onto a filter paper with gold electrodes. Details regarding material preparation and device fabrication are described in the experimental section. Figure 1f shows the corresponding optical microscope image of the device. It can be seen that the MXene/VP hybrid nanosheets fill up the channel and form a uniform film. The suction filtration process effectively avoids uneven aggregation and coffee ring effect, which is beneficial to the transport of carriers between nanosheets. The morphologies and structures of MXene and VP nanosheets were ascertained by TEM and Raman spectroscopy (Fig. S1). At the microscale, complex carrier dynamics processes such as intersheet hopping transport and interfacial trapping of photogenerated carriers in the heterojunction network composed of MXene and VP nanosheets lead to the PPC effect (Fig. 1b), thus enabling the device to act as an optoelectronic synapse. Figure 1g presents a SEM image of the hybrid film. It can be seen that MXene and VP nanosheets are interconnected or overlapped with each other, forming a heterojunction network.

The energy dispersive X-ray spectroscopy (EDS) elemental mapping confirms the homogeneous mixing of MXene and VP nanosheets (Fig. S2). At the nanoscale, a local van der Waals heterojunction with a built-in electric field is formed between adjacent MXene and VP nanosheets (Fig. 1c). The built-in electric field effectively promotes the separation of photogenerated carriers, and the excellent conductivity of MXene further improves the transport of carriers, thus significantly enhancing the photoresponse of VP. Meanwhile, gas molecules are easily adsorbed on the surface of 2D materials with large specific surface area, altering the optoelectronic response of the devices through the charge transfer with the nanosheets and the effects on the heterojunction barrier. Figure 1h is a TEM image of the hybrid film, from which a heterostructure of MXene/VP can be clearly found, exactly as envisioned in Fig. 1c. While at the atomic scale, the unique electronic structure of P atoms with a lone pair of electrons endows VP with high reactivity for gas adsorption (Fig. 1d) [30, 39], thereby facilitating olfactory perception.

### Analysis of Band Structure and Carrier Transfer

**Fig. 2:**
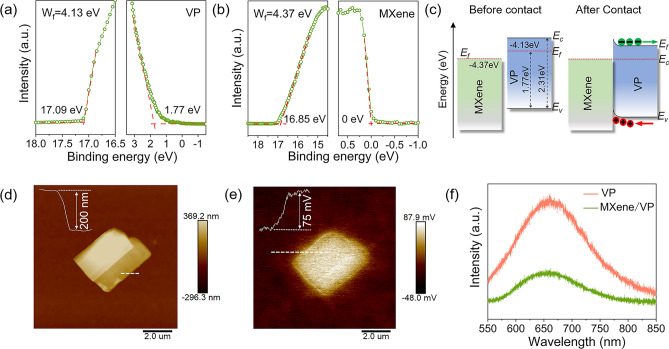
UPS spectra of **a** VP and **b** MXene. **c** Band structure diagram of MXene and VP before and after forming heterojunction. **d** The AFM image of the MXene/VP heterojunction. **e** KPFM image corresponding to **d**. **f** PL spectra of the pure VP and MXene/VP hybrid film upon excitation at 532 nm

**Fig. 3:**
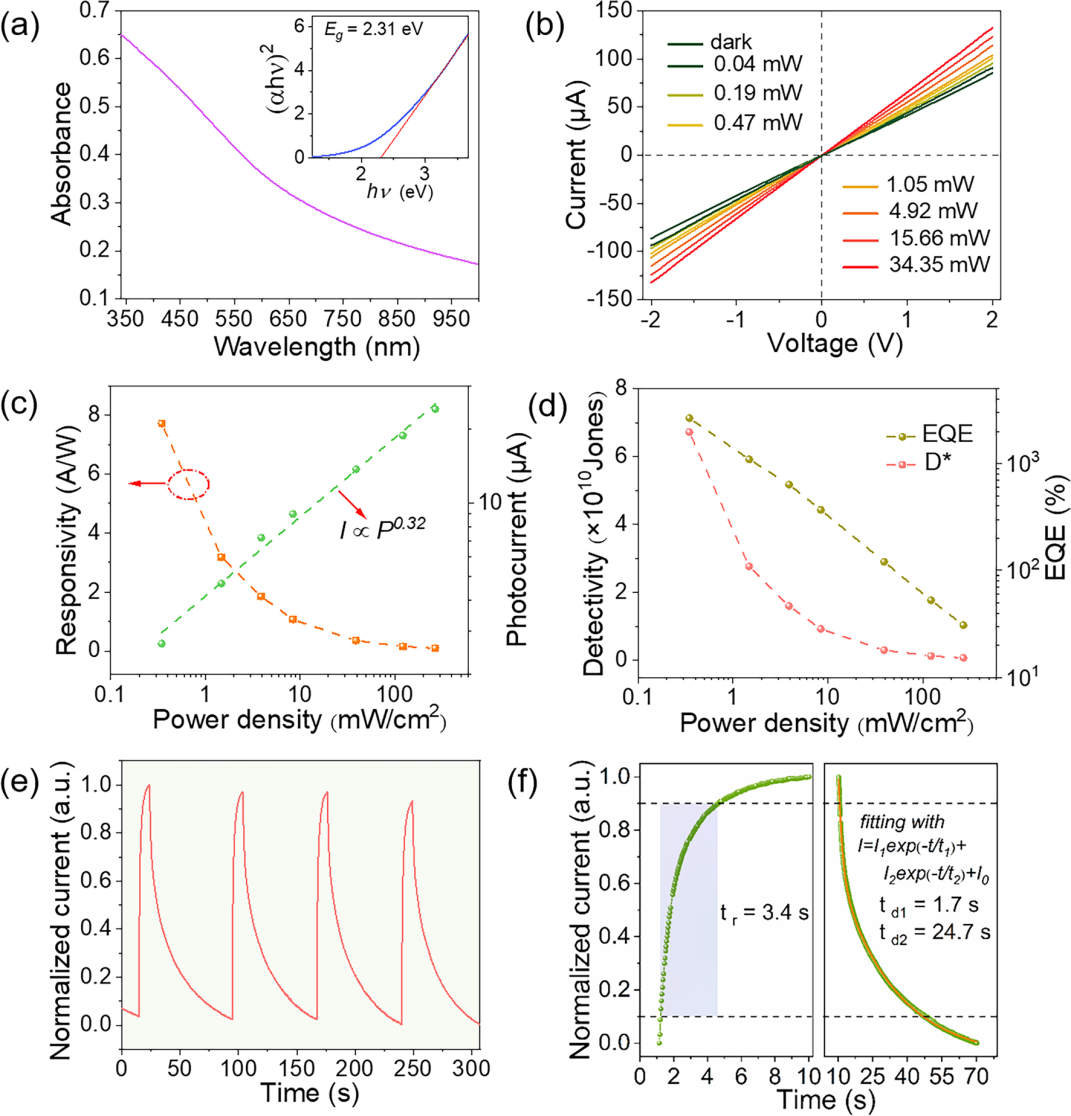
**a** Optical absorption spectrum of the VP nanosheets dispersion, inset is the calculated band gap of VP. **b** *I-V* curves of the MXene/VP heterojunction device under different light powers at 360 nm. **c** Photocurrent and responsivity, as well as **d** D^*^and EQE of the MXene/VP heterojunction device under different light power densities. **e** Time-resolved photoelectric response of the MXene/VP heterojunction device at 360 nm. **f** Rise and decay times of the MXene/VP heterojunction device

In order to analyze the energy band structure of the MXene/VP heterojunction and accordingly clarify the carrier transfer process, UPS, KPFM and steady-state PL spectroscopy tests were performed. Figure 2a and b shows the UPS spectra of VP and MXene. The work functions of VP and MXene can be obtained by subtracting the cut-off energy (17.09 and 16.85 eV) from the excitation energy (He I, 21.22 eV) as 4.13 and 4.37 eV, respectively. Furthermore, the difference between the Fermi level (*E*_*f*_) and the valence band top (*E*_*V*_) of VP is determined to be 1.77 eV by calculating the lowest binding energy, while that value of MXene is 0 eV due to its metallic nature. Combined with the band gap value of 2.31 eV of VP (Fig. 3a), we can depict the band structure diagram of MXene and VP before and after contact as shown in Fig. 2c. When they are assembled into van der Waals heterojunctions, the* E*_*f*_ tends to be the same, causing the conduction band (*E*_*C*_) of VP to bend upward toward MXene and form a Schottky junction. In the depletion region of the Schottky junction, there is a built-in electric field directed from VP to MXene. Driven by this built-in electric field, photogenerated holes in VP are injected into MXene under illumination, while electrons remain in VP. As a result, the separation and transport of photogenerated carriers are effectively promoted, which improves the photoelectric response of VP.

To further confirm the formation of local Schottky junctions, we measured the contact potential difference (CPD) between MXene and VP by KPFM. Note that in order to facilitate the positioning of the MXene/VP heterojunction, a layer of Mxene film was spin-coated on the sapphire substrate, and then VP nanosheets were spin-coated on the MXene film. Figure 2d shows the atomic force microscope (AFM) image of VP nanosheets on the surface of MXene film for the KPFM measurement. The height profile along the white dashed line shows that the thickness of the VP nanosheet is about 200 nm. The KPFM image in Fig. 2e shows the surface potential mapping corresponding to the heterojunction in Fig. 2d. It can be clearly seen that the surface potential of MXene is obviously lower than that of VP and there is a CPD of about 75 mV between them. This result indicates that the work function of MXene is greater than that of VP and there is a built-in electric field from VP to MXene, which is consistent with the UPS analysis. Figure 2f shows the PL spectra of pure VP and MXene/VP heterojunction. Pure VP exhibits a high PL intensity resulting from strong radiative recombination, but it is clearly quenched when VP forms heterojunctions with MXene, implying that the recombination of e–h pairs in VP is suppressed.

### Photoelectric Response Performance

As discussed above, the addition of MXene greatly improves the photoelectric response of VP, so we first investigated the photoelectric response of the hybrid film with a volume ratio of 0.02:1 (mL) of MXene and VP dispersions. Figure 3a shows the optical absorption spectrum of the VP nanosheet dispersion. The absorbance increases monotonically with decreasing wavelength, with obviously enhanced absorption below 600 nm. The inset shows that the optical band gap is calculated to be 2.31 eV by the Tauc plot method, which is close to the values reported in literatures [29, 31]. In view of the stronger absorption of VP in the UV band, we first tested the photoelectric response of the device to 360 nm UV light. Figure 3b shows the *I*-*V* curves in the dark and under illumination with different UV light powers. The current under illumination is greater than that in the dark at any bias voltage and increases continuously with the increase of light power. The photocurrent under different light power densities at 1 V is plotted in Fig. 3c, and their quantitative relationship can be fitted as a power law by Eq. (1):

1 $${I}_{ph}\propto {P}^{\alpha }$$

where *I*_*ph*_ is the photocurrent, *P* is the light power density and *α* (0 < *α* < 1) is the power index reflecting the linearity between the photocurrent and light power density. The fitted power index is 0.32, which deviates from the ideal value of 1. The deviation from the linear response is attributed to the complex process of carrier generation, transfer, trapping and recombination in the hybrid film [40, 41]. Responsivity is the core parameter to measure the photoelectric response level and can be calculated by Eq. (2):

2 $$R={I}_{ph}/(P\cdot S)$$

where *R* is the responsivity and *S* is the effective illumination area. As shown in Fig. 3c, the responsivity is negatively correlated with light power density with a maximum value of 7.7 A W^−1^.

In contrast, the pure VP device shows a photocurrent of only 100 pA at 10 V bias, and the responsivity was 0.36 μA W^−1^ (see Fig. S3), which was 7 orders of magnitude lower than that of the MXene/VP device. Even compared with pure VP devices based on Si substrates reported in literatures, the responsivity of our device is still 3–6 orders of magnitude higher [31, 33]. A detailed comparison of responsivity is shown in Fig. S4. Such a huge enhancement of the responsivity fully proves that forming heterojunctions with MXene can greatly improve the photoelectric response of VP. The specific detectivity (D^*^, reflecting the ability to detect weak light) and the external quantum efficiency (EQE, representing the ratio of collected carriers to incident photons) were also calculated by Eqs. (3) and (4):

3 $${D}^{*}=R\cdot \sqrt{S/(2\cdot q\cdot {I}_{d})}$$

4 $$EQE=(R\cdot h\cdot c)/(\lambda \cdot q)$$

where *q* is the elementary charge, *I*_*d*_ is the dark current, *h* is the Plank constant, *c* is the light velocity and *λ* is the wavelength. As shown in Fig. 3d, the D^*^ value calculated at the minimum light power density is 6.73 × 10^10^ Jones, which is comparable with previous reported 2D materials-based devices [42, 43]. The obtained EQE is as high as 2.67 × 10^3^%, which is attributed to the enhanced carrier separation and collection efficiency by MXene. We also investigated the dynamic response characteristics of the MXene/VP heterojunction device. Figure 3e shows the time resolved photoelectric response under intermittent UV illumination. The device demonstrates good repeatability and stability of photoswitching behavior. The rise (*t*_*r*_) and decay (*t*_*d*_) times can be obtained from a single switching cycle enlarged in Fig. 3e. As shown in Fig. 3f, the rise time is about 3.4 s, but it takes a relatively long time for the current to decay after turning off the UV illumination. The decay process can be well fitted by an exponential function with two relaxation times shown in Eq. (5):

5 $$I={I}_{1}\cdot exp(-t/{t}_{1})+{I}_{2}\cdot exp(-t/{t}_{2})+{I}_{0}$$

where *I*_*0*_, *I*_*1*_ and *I*_*2*_ are constants. The fitted time constants of fast component (*t*_*1*_) and slow component (*t*_*2*_) are 1.7 and 24.7 s, respectively. The PPC effect is associated with complex carrier dynamics, such as intersheet hopping transport, trapping and de-trapping at heterojunction interfaces and oxidation defects of VP surface (analyzed by XPS in Fig. S5) [33, 44]. The effect of different mixing ratios of MXene and VP on the photoelectric response performance has also been investigated, as discussed in detail in Fig. S6. Although the increase in the proportion of MXene will further improve the responsivity, the dark current will also increase significantly. Therefore, the device with a moderate ratio of MXene to VP (0.02:1) were selected for overall performance testing. In addition, it can be seen from Fig. 3a that VP also absorbs part of visible light, so we also studied the response characteristics of MXene/VP heterojunctions to visible light with a 532 nm laser as light source, the test results and discussion are shown in Fig. S7.

### Optoelectronic Synapse Characteristics

**Fig. 4:**
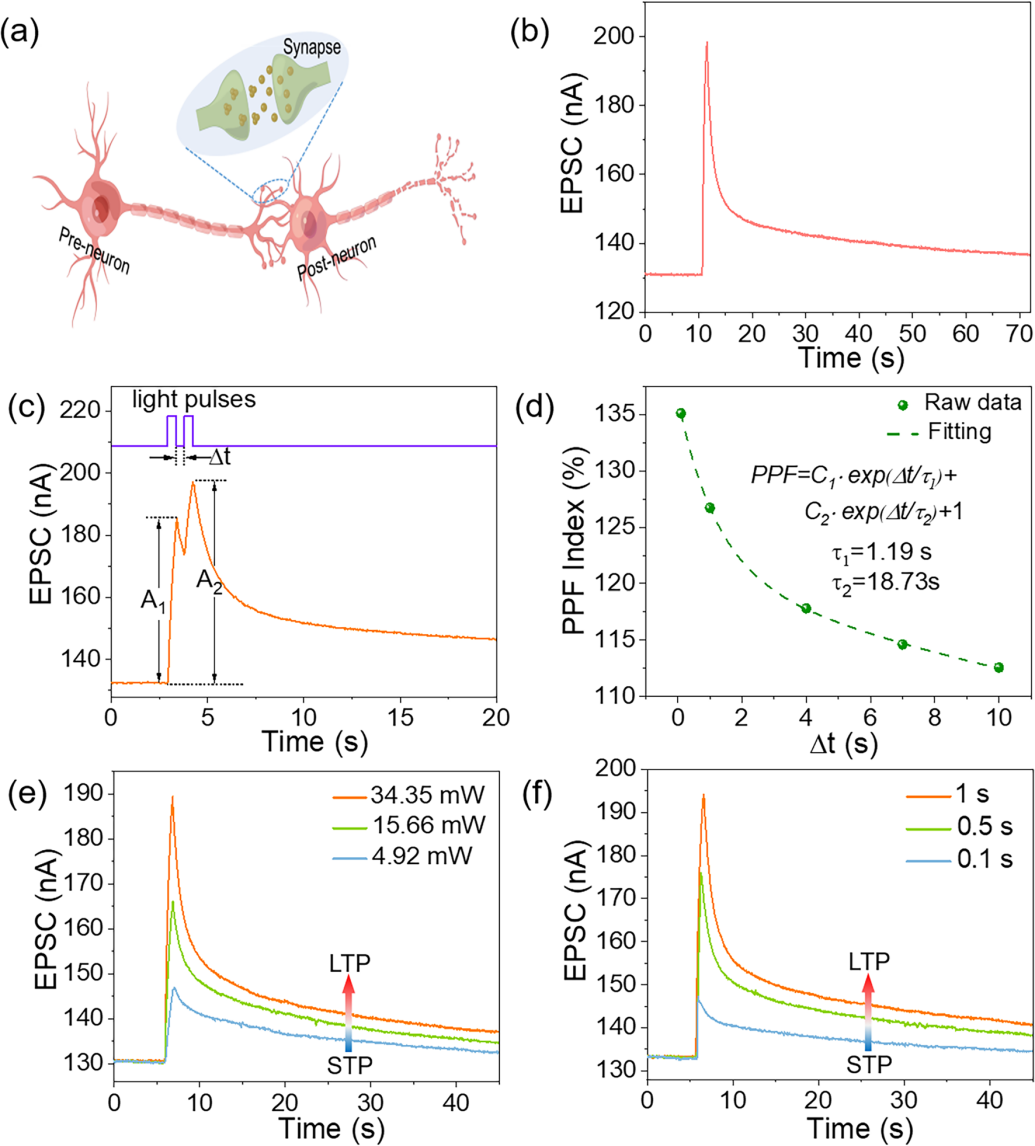
**a** Schematic diagram of biological neurons and synapses. **b** EPSC of the MXene/VP optoelectronic synapse triggered by a light pulse (360 nm, duration of 1 s). **c** EPSC triggered by a pair of light pulses with an interval time of Δ*t*. **d** PPF index as a function of the interval time of light pulse pairs. The transition from STP to LTP by increasing the light **e** pulse intensity and **f** pulse width

As above described, the PPC effect were observed in MXene/VP heterojunction device, which enables the device to function as an optoelectronic synapse to mimic visual perception and memory, with multiple characteristics of biological synapses such as excitatory postsynaptic currents (EPSC), paired-pulse facilitation (PPF), short-term plasticity (STP), long-term plasticity (LTP) and “learning-experience” behavior. Figure 4a schematically illustrates the biological synapse. Synapse acts as a communication site that transmits electrical or chemical signals between two neurons [45]. In our proof-of-concept MXene/VP optoelectronic synapse, UV light pulses are regarded as presynaptic stimulus and channel current as synaptic weight. An applied presynaptic spike (light pulse) can trigger an EPSC in the MXene/VP heterojunction channel at a small bias of 1 mV, as shown in Fig. 4b. The current decays rapidly and then slowly over time when the light is turned off, and is still 2.4% higher than the initial value after 100 s. This phenomenon is consistent with the decay process of postsynaptic potential in biological synapses [46]. When two consecutive presynaptic spikes are applied, the amplitude of the postsynaptic current will increase continuously, and the rate of increase (A_2_/A_1_) is defined as PPF, as shown in Fig. 4c. PPF is a typical manifestation of STP, which is closely related to the temporal recognition and decoding of visual signals in biological systems [22]. PPF strongly depends on the time interval (Δ*t*) between two presynaptic spikes, so we measured the PPF index (PPF index = A_2_/A_1_ × 100%) at different time intervals. As shown in Fig. 4d, the PPF index decreases from 135 to 112% as Δt increases from 0.1 to 10 s after applying light pulse pairs with a duration of 1 s. This is consistent with the fact that there is more recombination of the trapped carriers within the longer Δ*t*. The decay curve of the PPF exponent with Δ*t* matches well with the double exponential function shown in Fig. 4d, with relaxation times *τ*_*1*_ and *τ*_*2*_ of 1.19 and 18.73 s, respectively, comparable in scale to those of a biological synapse [47, 48].

**Fig. 5:**
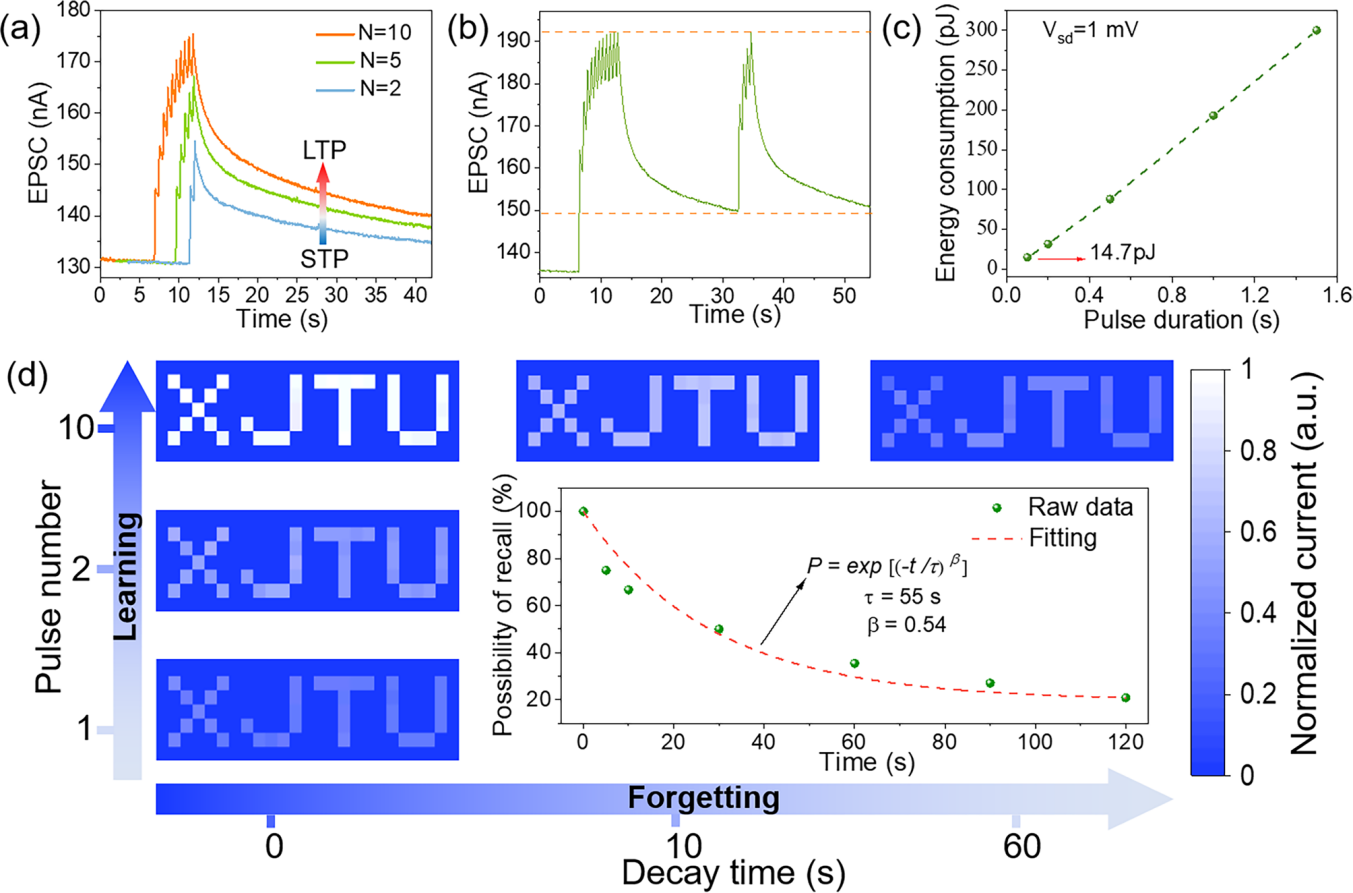
**a** The transition from STP to LTP by increasing the number of light pulses. **b** The “learning-experience” behavior of the MXene/VP optoelectronic synapse. **c** The energy consumption of the MXene/VP optoelectronic synapse. **d** Normalized EPSC mapping images with a “XJTU” letter pattern under increasing light pulse numbers (vertical axis) and decay time (horizontal axis). The inset is the image forgetting process fitted with the Ebbinghaus forgetting curve

In biological brains, the ability of synaptic weights to be modulated by neural activity is known as synaptic plasticity, which can be divided into STP and LTP according to the length of retention time and are an important bases for learning and memory [49]. Here, the transition from STP to LTP was also mimicked in the MXene/VP optoelectronic synapse by different stimulus modes. Figure 4e and f shows the EPSC curves under varying light pulse intensity and width. Similarly, with increase of the light pulse intensity (4.92 to 34.35 mW) and pulse width (0.1 to 1 s), both the amplitude and retention time of EPSC increase correspondingly, which is in analogy with the transition from STP to LTP in biological synapses [50]. In addition, the increase in the number of light pulses can also lead to the transition from STP to LTP (Fig. 5a), which is similar to the fact that frequent stimulation can increase the synaptic weight in biological synapses. Based on the biomimetic characteristics of the device, the “learning-experience” behavior is demonstrated, as shown in Fig. 5b. For our device, the light-on state corresponds to learning or relearning and the light-off state corresponds to the forgetting process. During the first learning process, the EPSC gradually increases when successive 10 light pulses are applied to the device, and then decays spontaneously after the light is removed. The EPSC retains 25% of the increment after 20 s, which means that the memory of the first learning is forgotten to 25%. In the second learning process, the EPSC returns to the maximum value by applying only 4 light pulses, and maintains a higher value than the first learning after the same decay time. This result corresponds to the fact that it takes less time to relearn forgotten information and that the memory will be enhanced after learning, surprisingly similar to the learning and memory behavior of the human brain [51]. Low power consumption is one of the fascinating advantages of biological brains, so we also consider the energy consumption of the MXene/VP optoelectronic synapse. The energy consumption for per synaptic event is defined as *I*_*peak*_ × *V* × *t*, where *I*_*peak*_, *V* and *t* refer to the peak value of the EPSC, the applied bias voltage and the pulse duration time, respectively. As shown in Fig. 5c, the energy consumption increases with the increase of the pulse duration time at 1 mV bias and the minimum energy consumption is 14.7 pJ when the pulse duration time is 0.1 s, which is lower than those reported 2D material-based optoelectronic synapses [52–55]. A more detailed comparison with reported optoelectronic synapses based on 2D materials and their heterojunctions is presented in Table S1.

Vision is the most important way for humans to obtain outside information, and thus visual memory is the most effective memory mode of the human brain. Artificial synapses for the construction of neuromorphic vision should exhibit a good brain-like visual memory behavior. To this end, the “XJTU” letter pattern composed of 7 × 25 pixels is used as the input signal to investigate the visual memory features of the MXene/VP photoelectric synapse through a single-pixel imaging system. Figure 5d shows the evolution of the pixel grayscale of the letter pattern with the number of light pulses and decay time. Note here that the pixel grayscale derived from the normalized EPSC represents memory strength, the pulse number corresponds to the number of learning times, and the decay process represents forgetting. It can be seen that the letter pattern after first study is not very clear due to the low memory strength, but the distinct target image can be recognized after 10 times of learning. Subsequently, the grayscale reduction of the image during the forgetting process was also recorded. As time goes by, the recognizability of the letter pattern gradually decreases, and the memory strength of the image is obviously weakened after 1 min. The Ebbinghaus forgetting curve illustrates how information is lost in the human brain over time, which can be expressed quantitatively by Eq. (6) [56]:

6 $$P={\text{exp}}[{(-t/\tau )}^{\beta }]$$

where *P* is the possibility of recall, *t* is time, and *τ* is the specific relaxation time and *β* is the exponent that ranges from 0 to 1. As shown in the inset of Fig. 5d, the forgetting process of the image fits the Ebbinghaus forgetting curve very well with the values of *τ* and *β* of 55 s and 0.54, which demonstrates the good brain-like learning and memory behavior of the MXene/VP optoelectronic synapse and its great potential in developing the human visual system.

### Visual-Olfactory Crossmodal Perception

**Fig. 6:**
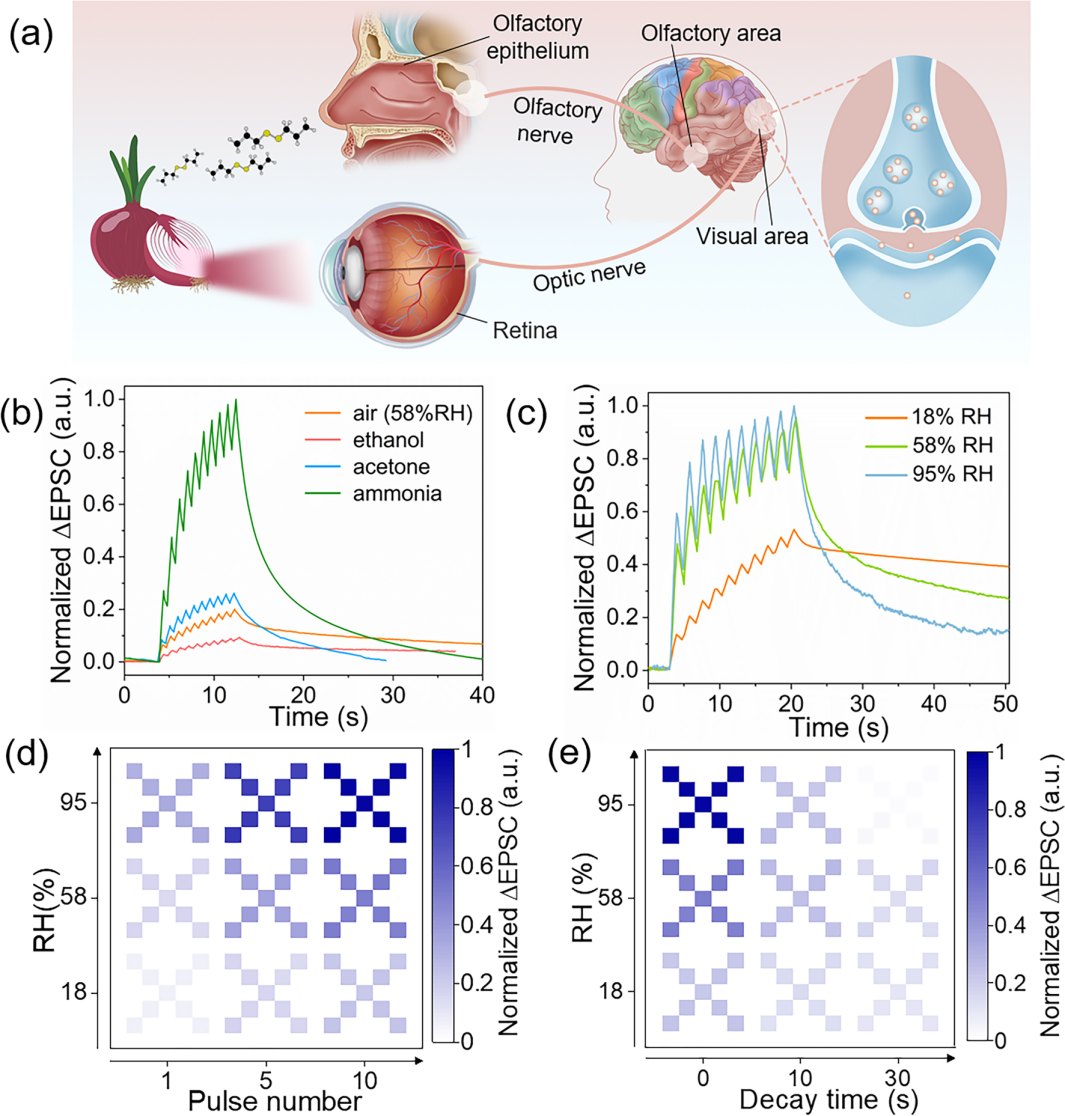
**a** Schematic diagram of crossmodal interaction between vision and olfaction in the human perceptual system. **b** ΔEPSCs triggered by 10 successive light pulses in environments with different gas atmospheres. **c** ΔEPSCs triggered by 10 successive light pulses in environments with different RH. The **d** recognition and **e** forgetting process of the letter “X” pattern in environments with different RH

The perception and memory processes of human are crossmodal, that is, the information input from different sense organs will interact in a synergistic or antagonistic manner, thereby strengthening the ability to perceive the environment or memory. For example, Tsushima et al. found that fragrance can change the human perception of the moving speed of pictures through psychological and physiological experiments [57]. Therefore, optoelectronic synapses with visual-olfactory crossmodal perception for developing biomimetic visual perception and memory are highly desired. Due to the excellent hydrophilicity of MXene, the large specific surface area of 2D materials and the unique electronic structure of VP, the gas atmosphere-dependent photoelectric response is expected for the MXene/VP optoelectronic synapse. Figure 6a illustrates the process of crossmodal interaction between vision and olfaction in the human perceptual system. Stimulation of specific odors during visual information input can result in enhancement or inhibition of visual perception. Figure 6b plots the EPSC curves stimulated by 10 UV pulses in different gas environments. It can be seen that amplitudes and decay times of the postsynaptic currents in different odor environments are distinct under the same visual stimulus, which is analogous to the visual-olfactory crossmodal perception process of human. The mechanism should be attributed to the different photoelectric gas-sensing processes of VP for various gases. Taking ammonia as an example, ammonia molecules with lone electrons are adsorbed on the surface of VP and act as electron donors to release electrons to conduction band of VP, which increases the carrier density and shrinks the depletion regions of the Schottky junctions, thereby reducing the resistance of the hybrid film and increase the photocurrent. In order to quantitatively explore the differences in visual perception in environments with different odor levels, the photoelectric responses were tested in environments with different relative humidity (RH), where different concentrations of water molecules were regarded as different odor levels. Figure S8 shows the variation of the baseline (dark current) of EPSCs with the RH in the absence of light. The RH has a significant inhibitory effect on dark current, because water molecules adsorbed on the surface of the nanosheets of the hybrid film expands the intersheet spacing, thereby increasing the tunneling resistance [58, 59]. This interesting phenomenon inspires us that the inhibitory postsynaptic current (IPSC) can also be obtained in the MXene/VP optoelectronic synapse by applying humidity pulses, as usually this is difficult to achieve in planar two-terminal synaptic devices due to the absnce of gate [60–62]. As shown in Fig. S9, the long-term depression (LTD), a common and indispensable behavior in biological synapses, can also be achieved in the MXene/VP optoelectronic synapse by successive humidity pulses. Accordingly, LTP/LTD synaptic behaviors with light potentiation and humidity depression, which are normally only available in three-terminal synaptic devices, can also be realized in our two-terminal synaptic device, as shown in Fig. S10. In addition, the effect of the RH on the amplitude and decay time of the EPSC was also investigated. Figures S11 and 6c show the EPSC curves under the excitation of a single light pulse and 10 light pulses in different RH environments, respectively. Interestingly, the ΔEPSC (peak value minus baseline) increases but decay time decreases with increasing RH.

The increase of ΔEPSC is attributed to the reduction of dark current, while the reduced decay time may be related to the fact the H^+^ and OH^−^ produced by the dissociation of water molecules combine with electrons and holes, accelerating the disappearance process of the trapped photogenerated carriers. Figure 6d and e maps the recognition and forgetting processes of the letter “X” in environments with different RH. It can be seen that the higher the RH (that is, the higher odor level), the more rapidly the EPSC increases but also the faster it decays. If we still assumed that the pixel grayscale represents the memory strength, we can conclude a typical visual-olfactory crossmodal perception behavior of this photoelectric synapse, that is, visual information is perceived more strongly in environments with high odor levels, but is also forgotten more quickly over time after the visual stimulus is removed. This phenomenon is just like the memory behavior of the human brain discussed in Bjork's memory theory—retrieval strength of memory is negatively correlated with storage strength [63, 64]. This is the first report of such complex memory behavior in optoelectronic synapses and demonstrates the bright application prospects of MXene/VP heterojunctions in crossmodal neuromorphic vision sensors.

## Conclusions

In summary, we have successfully developed an optoelectronic synapse with visual-olfactory crossmodal perception based on MXene/VP van der Waals heterojunctions. The hybrid film composed of MXene/VP heterojunction network prepared by a simple solution process exhibit a far superior photoelectric response than pure VP, with a high responsivity of 7.7 A W^−1^. Subsequently, an optoelectronic synapse with multiple synaptic behaviors such as EPSC, PPF, STP, LTP and “learning-experience” behavior is demonstrated. Furthermore, we prove that the optoelectronic synapse exhibits distinct behaviors in environments with different gas atmospheres, enabling it to process visual-olfactory crossmodal perception. This work reports the first VP-based optoelectronic synapse for neuromorphic crossmodal perception, demonstrating the promising prospects of VP for optoelectronic devices and providing inspiration for the development of optoelectronic synapses with crossmodal perception.

## Supplementary Information

Below is the link to the electronic supplementary material.

Supplementary file1 (PDF 840 KB)
