# Supplementary material for: Optoelectronic Synapses Based on MXene/Violet Phosphorus van der Waals Heterojunctions for Visual-Olfactory Crossmodal Perception
Source: Nanomicro Lett. 2024 Feb 1;16:104. doi: 10.1007/s40820-024-01330-7 (PMC10834395; doi:10.1007/s40820-024-01330-7)
Supplement: Supplementary file 1 — Supplementary file1 (PDF 840 KB) [file 40820_2024_1330_MOESM1_ESM.pdf]

Supporting Information for

Optoelectronic Synapses Based on MXene/Violet Phosphorus van der Waals Heterojunctions for Visual-Olfactory Crossmodal Perception

Hailong Ma[1], Huajing Fang[1,]*, Xinxing Xie[1], Yanming Liu[2], He Tian[2,]*, Yang Chai[3,]*

[1] Center for Advancing Materials Performance from the Nanoscale (CAMP-Nano), State Key Laboratory for Mechanical Behavior of Materials, Xi'an Jiaotong University, Xi'an 710049, P. R. China

[2] Institute of Microelectronics and Beijing National Research Center for Information Science and Technology (BNRist), Tsinghua University, Beijing 100084, P. R. China

[3] Department of Applied Physics, The Hong Kong Polytechnic University, Hong Kong, P. R. China

*Corresponding authors. E-mail: fanghj@xjtu.edu.cn (Huajing Fang); tianhe88@tsinghua.edu.cn (He Tian); ychai@polyu.edu.hk (Yang Chai)

Supplementary Figures and Tables

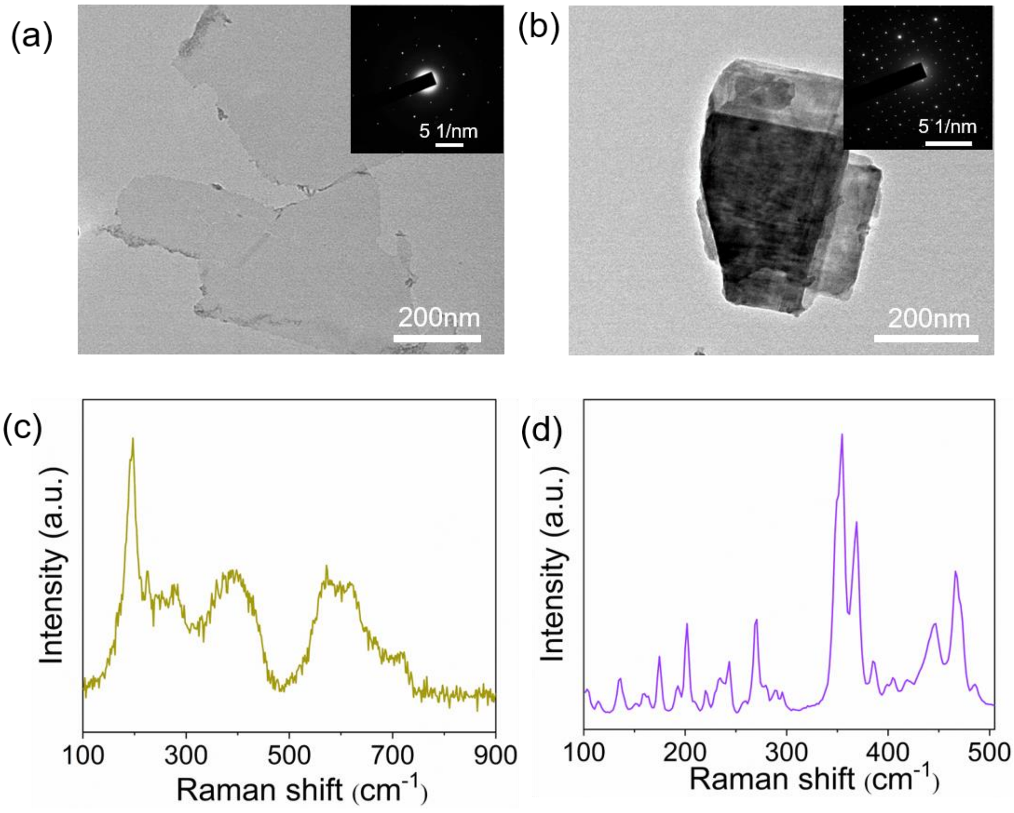

Fig. S1 TEM images of **a** MXene and **b** VP nanosheets. Insets show the SAED patterns. The Raman spectra of **c** MXene and **d** VP

The TEM images and selected area electron diffraction (SAED) patterns in Fig. S1a, b confirms that both MXene and VP have typical 2D sheet-like morphology and good crystallinity. The Raman spectrum of MXene (Fig. S1c) exhibits three broad bands due to the coexistence of multiple surface functional groups [S1], and that of VP (Fig. S1d) is sharp and complex, which is derived from the good crystallinity and complex crystal structure of VP, with up to 84 atoms in a single unit cell [S2].

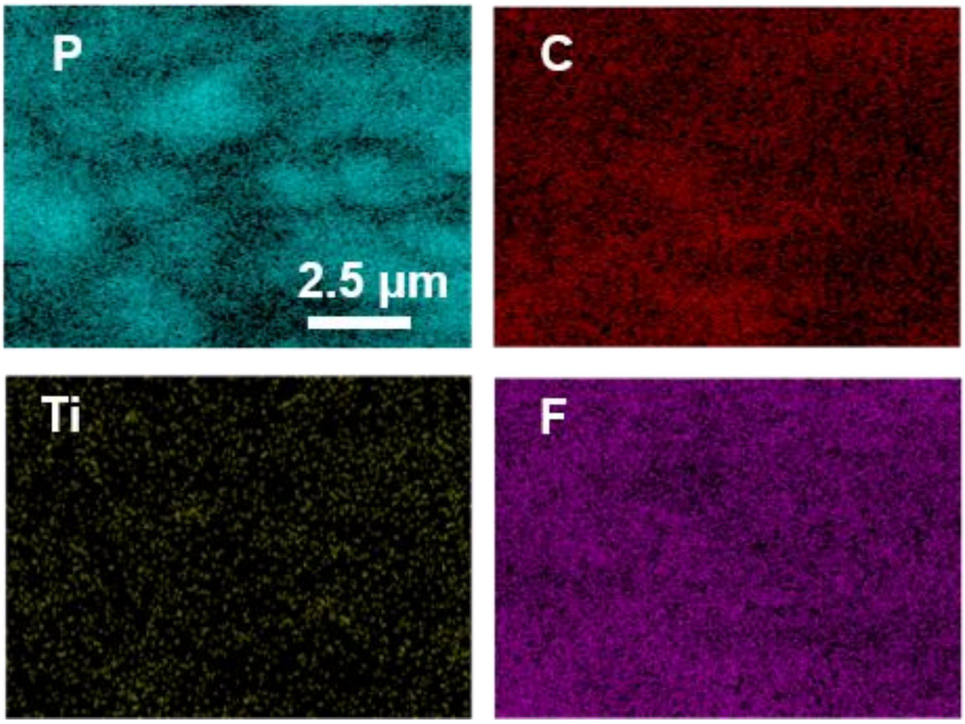

Fig. S2 EDS elemental mapping of the MXene/VP hybrid film corresponding to the SEM image in Fig. 1f

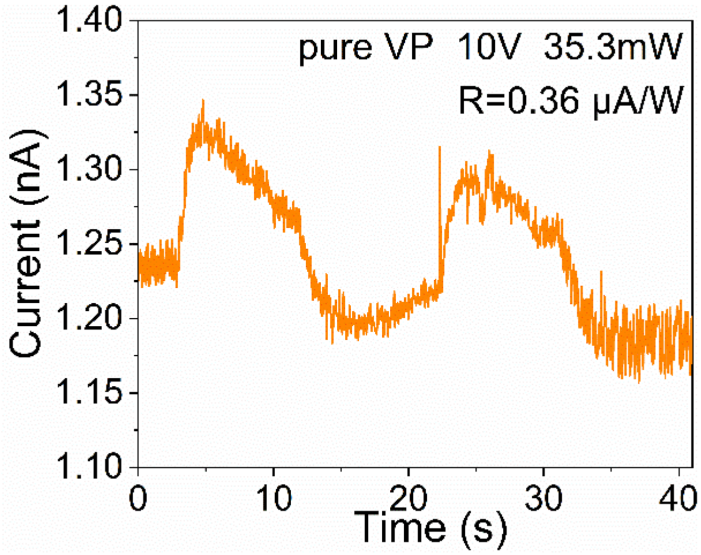

Fig. S3 Photoelectric response of pure VP

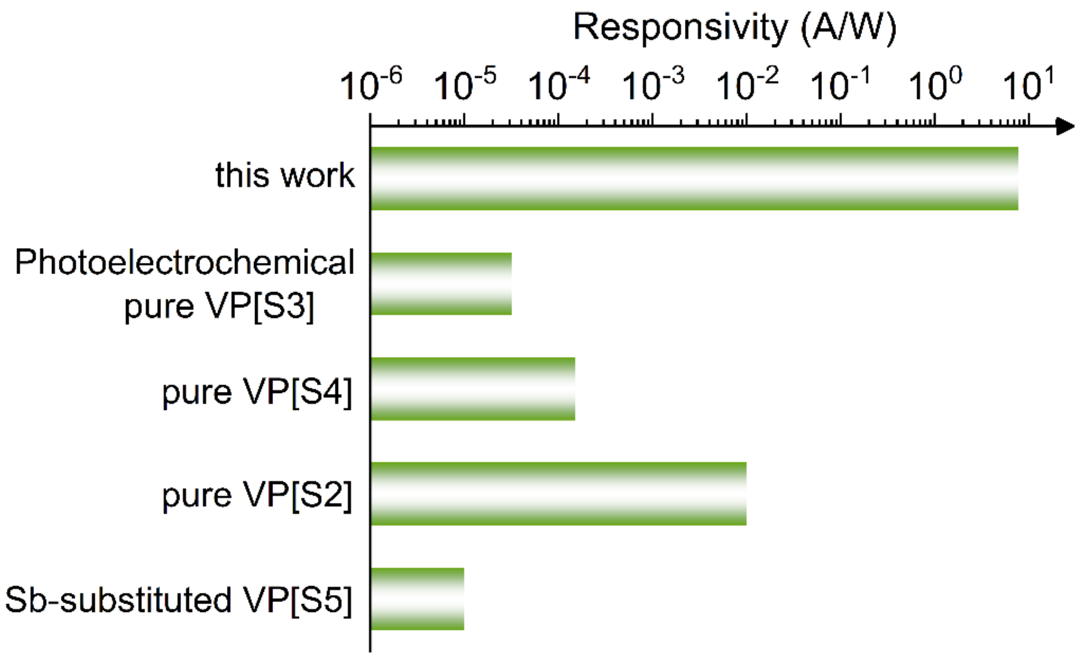

Fig. S4 Comparison of responsivity of VP-based photodetectors

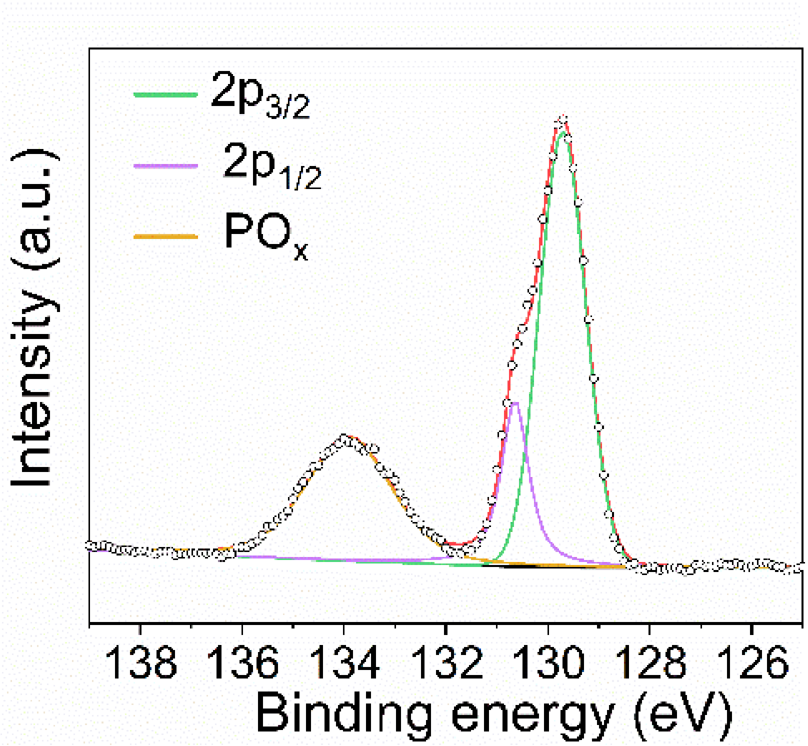

Fig. S5 The XPS spectrum of VP

The XPS peaks at 130.6 and 129.7 eV can be respectively assigned to P $2p_{1/2}$ and P $2p_{3/2}$, while the peak at 133.9 eV indicates the presence of phosphorus oxides on the surface of VP [27].

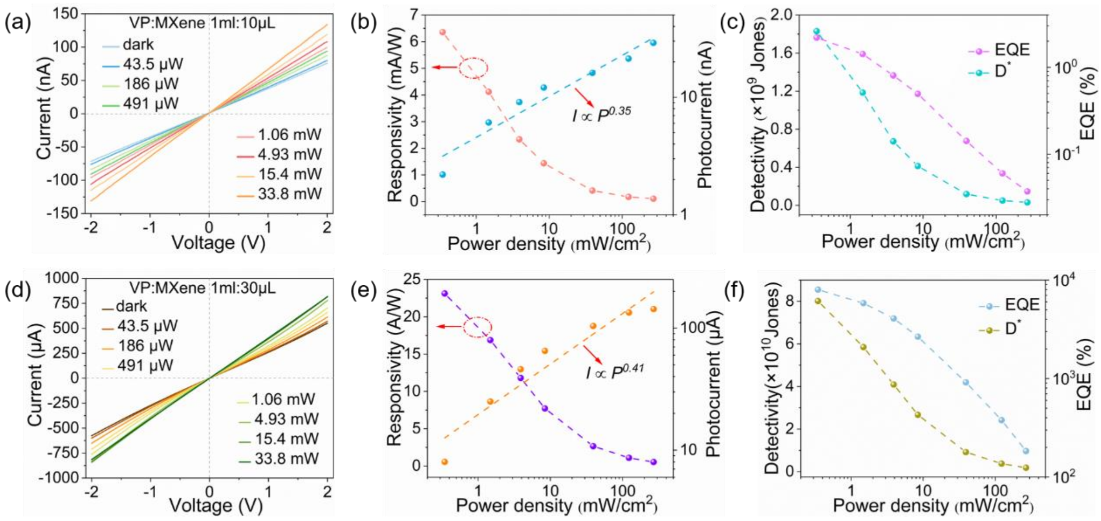

Fig. S6 Effect of mixing ratio of MXene and VP on photo response performance. **a** *I-V* curves, **b** responsivity and photocurrent, **c** EQE and D* of the device with a 0.01:1 volume ratio of MXene to VP at 360 nm. **d** *I-V* curves, **e** responsivity and photocurrent, **f** EQE and D* of the device with a 0.03:1 volume ratio of MXene to VP at 360 nm

Figure S6 shows the effect of the mixing ratio of MXene and VP on the photoelectric response performance. It can be found that as the proportion of MXene increases, the photocurrent, responsivity, EQE and D* of the device are all improved, with the maximum responsivity of 23.1 A/W at a volume ratio of MXene to VP of 0.03:1. These results undoubtedly indicate that forming heterojunctions with MXene can effectively improve the photoelectric response of VP. However, the disadvantage of increasing the proportion of MXene is that the dark current of the device will be significantly increased due to the excellent conductivity of MXene. The dark current increases dramatically from 37 nA to 260 μA when the ratio of MXene to VP is increased from 0.01:1 to 0.03:1. Therefore, we chose a moderate ratio of MXene to VP of 0.02:1 in the overall performance tests of the device.

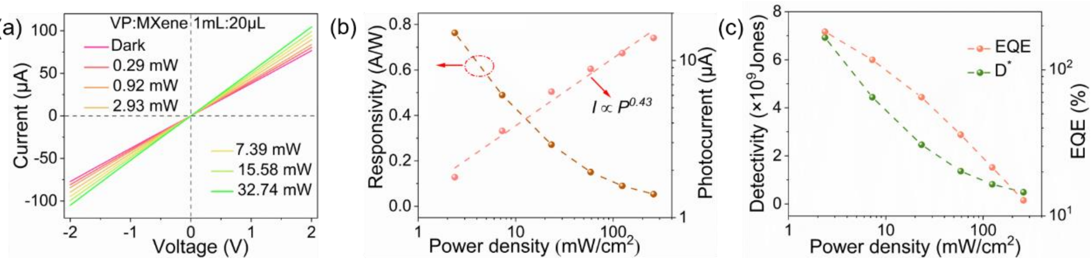

Fig. S7 a *I-V* curves, **b** responsivity and photocurrent, **c** EQE and D* of the device with a 0.02:1 volume ratio of MXene to VP at 532 nm

Figure S7 shows the response characteristics of the MXene/VP heterojunctions to visible light with a wavelength of 532 nm. *I-V* curves indicate that the MXene/VP heterojunctions have an obvious photoelectric response to 532 nm light. The maximum values of responsivity, EQE and D* are 0.76 A/W, 182% and 6.92 × 10^9 Jones respectively, which are lower than those values to 360 nm UV light. These results are consistent with the

optical absorption properties of VP shown in Fig. 3a and indicate the UV-Vis broadband photoelectric response characteristics of the MXene/VP heterojunctions.

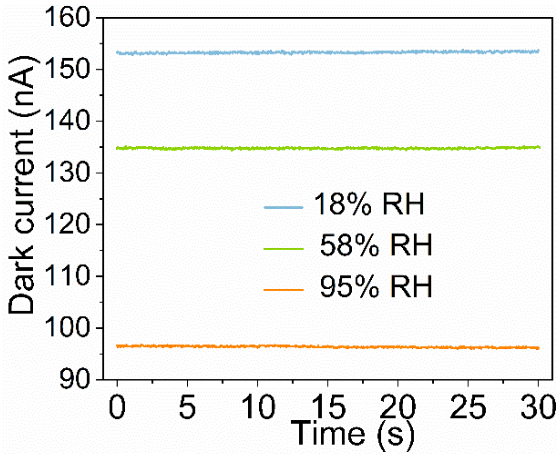

Fig. S8 Dark currents of the MXene/VP optoelectronic synapse in environments with different RH

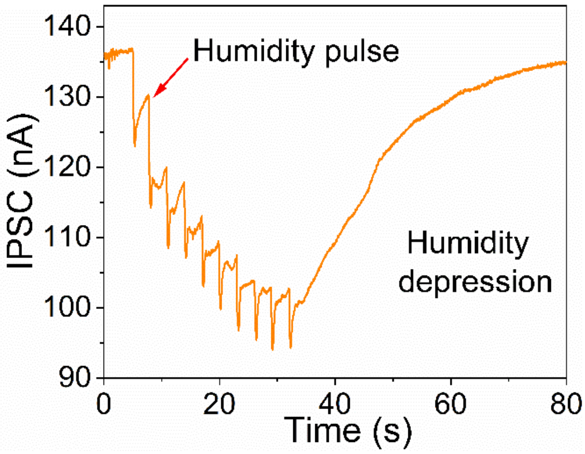

Fig. S9 Inhibitory postsynaptic current (IPSC) curve stimulated by 10 consecutive humidity pulses

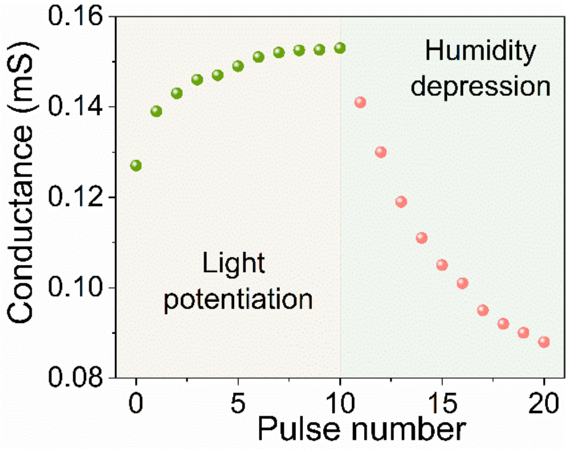

Fig. S10 Light potentiation and humidity depression in the MXene/VP optoelectronic synapse

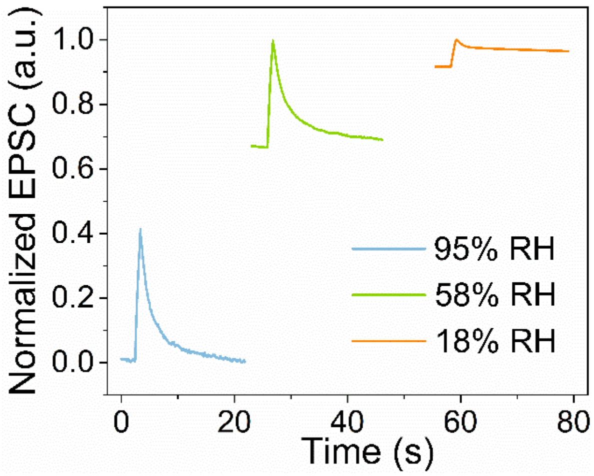

Fig. S11 The EPSC curve under the excitation of a single light pulse in environments with different RH

Table S1 Comparison of optoelectronic synapses based on 2D materials and their heterojunctions

Material (device structure	Synaptic functions	V_{sd} (V)	PPF (%)	inde	Energy consumption(p J)	Application	Refs.
Graphdiyne/Grap hene/PbS QD (2T)	EPSC/IPSC, PPF	0.01	180		–	Pattern recognition/In age memory	[S6]
Black Phosphoru (2T)	EPSC/IPSC, STDP, PPF	–	280		–	Logic computation	[S7]
α-In_2Se_3/GaSe (3T)	EPSC/IPSC, PPF, SNDP	0.5	121		–	Pavlov's dog	[S8]
ReS_2/h-BN (2T)	EPSC, PPF	–	132		1.2×10^7	Convolutiona neural networl	[S9]
MoS_2/h-BN (3T	EPSC/IPSC, PPF, SADP, SRDP	0.05	–		–	–	[S10]
ZnO nanosheet (2T)	EPSC, PPF, STDP, SRDP, SDDP, Learning-experience	0.05	170		87.5	Pavlov's dog	[S11]
Graphdiyne /Graphene (3T)	EPSC/IPSC, PPF, SRDP	0.01	163		650	Pattern recognition/Lo gic computation	[S12]
$CsPbBr_3/MoS_2$ (3T)	EPSC, PPF, SRDP, SDDF	0.1	–		4200	Pavlov's dog	[S13]
2D MOF (2T)	EPSC, PPF, SRDP	1	125		–	–	[S14]
MXene/VP (2T)	EPSC/IPSC, PPF, SADP, SNDP, SDDP Learning-experience	0.001	135		14.7	Image memory/ Crossmodal perception	This work

EPSC: Excitatory postsynaptic currents; IPSC: Inhibitory postsynaptic current; PPF: Paired-puls facilitation; SADP: Spike-amplitude-dependent plasticity; SNAP: Spike-number-depender plasticity; SDDP: Spike-duration-dependent plasticity; SRDP: Spike-rate-dependent plasticity; 2/3T 2/3 terminal; V_{sd}: Source-drain voltage; QD: Quantum dot; MOF: Metal-organic framework

Supplementary References

[S1] Ren, J. Zou, H. Lai, Y. Huang, L. Yuan et al., Direct laser-patterned MXene–perovskite image sensor arrays for visible-near infrared photodetection. Mater. Horiz. **7**(7), 1901–1911 (2020). https://doi.org/10.1039/d0mh00537a

[S2] A.G. Ricciardulli, Y. Wang, S. Yang, P.Samorì, Two-dimensional violet phosphorus: A p-type semiconductor for (opto)electronics. J. Am. Chem. Soc. **144**(8), 3660–3666 (2022). https://doi.org/10.1021/jacs.1c12931

[S3] L. Jin, R. Guo, T. Han, R. Wang, Y. Zhang, Ultrathin 2D violet phosphorus nanosheets: Facile liquid-phase exfoliation, characterization, and photoelectrochemical application. Adv. Funct. Mater. **33**(27), 2213583 (2023). https://doi.org/10.1002/adfm.202213583

[S4] Y. Li, S. Cai, W.K. Lai, C. Wang, L. Rogée et al., Impurity-induced robust trionic effect in layered violet phosphorus. Adv. Opt. Mater. **10**(1), 2101538 (2022). https://doi.org/10.1002/adom.202101538

[S5] F. Baumer, Y. Ma, Y. Liu, D. Pfister, T. Nilges et al., Synthesis, characterization, and device application of antimony-substituted violet phosphorus: A layered material. ACS Nano **11**(4), 4105–4113 (2017). https://doi.org/10.1021/acsnano.7b00798

[S6] Y.-X. Hou, Y. Li, Z.-C. Zhang, J.-Q. Li, D.-H. Qi et al., Large-scale and flexible optical synapses for neuromorphic computing and integrated visible information sensing memory processing. ACS Nano **15**(1), 1497–1508 (2021). https://doi.org/10.1021/acsnano.0c08921

[S7] T. Ahmed, S. Kuriakose, S. Abbas, M.J.S. Spencer, M.A. Rahman et al., Optoelectronics: Multifunctional optoelectronics via harnessing defects in layered black phosphorus. Adv. Funct. Mater. **29**(39), 1970272 (2019). https://doi.org/10.1002/adfm.201970272

[S8] F. Guo, M. Song, M.-C. Wong, R. Ding, W.F. Io et al., Multifunctional optoelectronic synapse based on ferroelectric van der waals heterostructure for emulating the entire human visual system. Adv. Funct. Mater. **32**(6), 2108014 (2022). https://doi.org/10.1002/adfm.202108014

[S9] S. Seo, J.-J. Lee, R.-G. Lee, T.H. Kim, S. Park et al., An optogenetics-inspired flexible van der waals optoelectronic synapse and its application to a convolutional neural network. Adv. Mater. **33**(40), 2170316 (2021). https://doi.org/10.1002/adma.202170316

[S10] M. Xu, T. Xu, A. Yu, H. Wang, H. Wang et al., Optoelectronic synapses based on

photo-induced doping in MoS_2/h-BN field-effect transistors. Adv. Opt. Mater. **9**(20), 2100937 (2021). https://doi.org/10.1002/adom.202100937

[S11] V. Krishnamurthi, T. Ahmed, M. Mohiuddin, A. Zavabeti, N. Pillai et al., A visible-blind photodetector and artificial optoelectronic synapse using liquid-metal exfoliated ZnO nanosheets. Adv. Opt. Mater. **9**(16), 2100449 (2021). https://doi.org/10.1002/adom.202100449

[S12] Z.-C. Zhang, Y. Li, J.-J. Wang, D.-H. Qi, B.-W. Yao et al., Synthesis of wafer-scale graphdiyne/graphene heterostructure for scalable neuromorphic computing and artificial visual systems. Nano Res. **14**, 4591–4600 (2021). https://doi.org/10.1007/s12274-021-3381-4

[S13] Y. Cheng, H. Li, B. Liu, L. Jiang, M. Liu et al., Neuromorphic photoelectric devices: Vertical 0D-perovskite/2D-MoS_2 van der waals heterojunction phototransistor for emulating photoelectric-synergistically classical Pavlovian conditioning and neural coding dynamics. Small **16**(45), 2070244 (2020). https://doi.org/10.1002/smll.202070244

[S14] Y. Liu, Y. Wei, M. Liu, Y. Bai, G. Liu et al., Two-dimensional metal-organic framework film for realizing optoelectronic synaptic plasticity. Angew. Chem. Int. Ed. **60**(32), 17440–17445 (2021). https://doi.org/10.1002/anie.202106519
